# Screening the NCI diversity set V for anti-MRSA activity: cefoxitin synergy and LC-MS/MS confirmation of folate/thymidine biosynthesis inhibition

**DOI:** 10.1128/spectrum.00541-23

**Published:** 2023-10-27

**Authors:** Shivani Gargvanshi, Gioia Heravi, Navid J. Ayon, William G. Gutheil

**Affiliations:** 1 Division of Pharmacology and Pharmaceutical Sciences, School of Pharmacy, University of Missouri-Kansas City, Kansas City, Missouri, USA; University of Calgary, Calgary, Alberta, Canada

**Keywords:** library screening, *Staphylococcus aureus*, microsome, P450, metabolism, drug discovery, chemical diversity, synergy, antibiotic drug resistance, MRSA, LC-MS/MS, metabolomics

## Abstract

New antibacterial agents and agent combinations are urgently needed to combat antimicrobial resistance. A multidimensional chemical library screening strategy was used to identify compounds in the National Cancer Institute (NCI) diversity set V library (1,593 compounds) with anti-methicillin-resistant *Staphylococcus aureus* (MRSA) activity. In this effort, library compounds were screened for anti-MRSA activity in both their original [un-metabolized (UM)] and human liver microsome-metabolized [post-metabolized (PM)] forms and in the absence and presence of sub-minimum inhibitory concentration (MIC) levels of cefoxitin. This strategy allows for the identification of intrinsically active agents, agents with active metabolites, and agents that can act synergistically with cefoxitin. Sixteen UM compounds with MICs ≤ 12.5 µM were identified. No agents with substantially enhanced activity after microsomal metabolism were found. Several agents showed significant apparent synergy with cefoxitin, and checkerboard assays were used to confirm synergy for four of these (celastrol, porfiromycin, 4-quinazolinediamine, and teniposide). A follow-up comparative screen in the absence and presence of 4-µM thymidine was used to identify three agents as likely folate/thymidine biosynthesis inhibitors. A liquid chromatography–mass spectrometry (LC-MS/MS) assay for deoxythymidine triphosphate (dTTP) was used to confirm these three as suppressing dTTP biosynthesis in MRSA. Bactericidal vs bacteriostatic activity was also evaluated. This study further demonstrates the utility of comparative library screening to identify novel bioactive agents with interesting synergies and biological activities. The identification of several folate/thymidine biosynthesis inhibitors from this small screen indicates that this pathway is a viable target for new drug discovery efforts.

**IMPORTANCE:**

New antibacterial agents are urgently needed to counter increasingly resistant bacteria. One approach to this problem is library screening for new antibacterial agents. Library screening efforts can be improved by increasing the information content of the screening effort. In this study, we screened the National Cancer Institute diversity set V against methicillin-resistant *Staphylococcus aureus* (MRSA) with several enhancements. One of these is to screen the library before and after microsomal metabolism as means to identify potential active metabolites. A second enhancement is to screen the library in the absence and presence of sub-minimum inhibitory concentration levels of another antibiotic, such as cefoxitin in this study. This identified four agents with synergistic activity with cefoxitin out of 16 agents with good MRSA activity alone. Finally, active agents from this effort were counter-screened in the presence of thymidine, which quickly identified three folate/thymidine biosynthesis inhibitors, and also screened for bactericidal vs bacteriostatic activity.

## INTRODUCTION

Antimicrobial resistance (AMR) in pathogenic bacteria is a major public health threat (1 – 4). Methicillin-resistant *Staphylococcus aureus* (MRSA) causes both nosocomial and community-acquired infections (5, 6). It is resistant to most β-lactam antibiotics including methicillin, oxacillin, amoxicillin, and cefoxitin and to many other antibiotic classes and agents (7). Chemical library screening is a popular drug discovery approach where hundreds to many thousands of compounds are screened in a high-throughput fashion to identify novel pharmacological and biological activities (8). Given that the emergence of resistance to single agents has so far proven inevitable, methods to reverse or prevent the emergence of resistance, such as the development of antibacterial agent combinations, seem essential (9 – 11).

In a prior study, a dimensionally enhanced library screening approach was demonstrated for screening a Food and Drug Administration (FDA)-approved drug library against MRSA (12). This approach uses added dimensions (human liver microsome metabolized library compounds and ±cefoxitin screening) to a standard library screen to provide valuable additional information while also providing a degree of screening redundancy. In this study, a variation of this approach was applied to MRSA using a non-FDA-approved library to assess the ability of this approach to identify interesting lead compounds in a general chemical (non-FDA) library screen. The National Cancer Institute (NCI) diversity set V library was used for this effort, which consists of 1,593 compounds selected to cover a wide range of chemical and pharmacophore space. This effort identified agents with good intrinsic anti-MRSA activity and agents with synergistic activity with cefoxitin. No agents with active metabolites were identified in this screen.

A key bottleneck in whole-cell screening for antibacterial activity is the determination of the mechanism of actions (MOAs) of newly identified agents (13, 14). Two of the compounds identified in this screen had obvious similarity to trimethoprim (diaminopyrimidine), a folate reductase inhibitor. Added thymidine, a key metabolite dependent on folate biosynthesis, is known to rescue *S. aureus* from folate/thymidine biosynthesis inhibition, including from both sulfamethoxazole and trimethoprim (15). A ±thymidine follow-up screen was therefore implemented, which identified three prospective folate/thymidine biosynthesis inhibitors—two obvious diaminopyrimidine-containing candidates plus a fluorinated pyrimidine compound similar to 5-fluorouracil. To provide further confirmation, their effect on bacterial deoxythymidine triphosphate (dTTP) pool levels was determined by liquid chromatography–mass spectrometry (LC-MS/MS) analysis. Compounds were also evaluated for bactericidal vs bacteriostatic activity, and spectrum of activity data against a panel of MRSA strains was used to identify agents with general activity against MRSA.

## RESULTS AND DISCUSSION

### Library screening and hit MIC determinations

Library screening was performed at 200 µM [nominal concentration for the post-metabolized (PM) library screen] as described in detail previously (12, 16). Following library screening, a pooled hit list was made [i.e., any compound that gave a hit (was active in suppressing bacterial growth) in any of the four un-metabolized (UM)/PM vs ±Cef screens was added to the list] for follow-up minimum inhibitory concentration (MIC) determinations. MICs for all the compounds in this pooled hit list were then determined by serial dilution in steps of two starting at 100 µM under all four screening conditions (UM − Cef, UM + Cef, PM − Cef, and PM + Cef) to give a final table of MICs. The results from these MIC determinations for minimum MICs of ≤12.5 µM are summarized in Table 1 and for all screening hits in Table S1. All inactive screened compounds are listed in Table S2. Celastrol is also included in Table 1 even though it had relatively weak activity since it showed significant apparent synergy with cefoxitin as discussed further below.

**TABLE 1:** MICs (µM) for top 14 NCI diversity set V compounds against MRSA (ATCC #43300)^*e*^

		UM^*f*^		PM^*g*^						
Name	PubChem CID^*h*^	Cef	+Cef	Cef	+Cef	Min_MIC	L2 (±Cef) *^a^*	AL2 (±Cef) *^b^*	L2 (UM/PM) *^c^*	AL2 (UM/PM) *^d^*
Clorobiocin	54677920	0.10	0.10	0.10	0.049	0.049	0	0.5	0	0.5
4-QDA^*i*^	16682542	0.39	0.10	25	25	0.10	**2**	1.0	**6**	**7.0**
Ethyl violet	16955	1.6	0.78	25	12.5	0.78	1	1.0	**4**	**4.0**
Bactobolin	54676871	3.1	1.6	12.5	6.25	1.6	1	1.0	**2**	**2.0**
Hitachimycin	54598584	1.6	1.6	3.1	3.1	1.6	0	0.0	1	1.0
NSC367428	339703	3.1	3.1	50	25	3.1	0	0	**4**	**3.5**
Porfiromycin	244989	12.5	3.1	25	25	3.1	**2**	1.0	1	**2.0**
Teniposide	54610154	12.5	3.1	25	12.5	3.1	**2**	1.5	1	1.5
Naphtanilide LB	67238	6.25	6.25	25	12.5	6.25	0	0.5	**2**	1.5
NSC207895	42640	6.25	6.25	200	200	6.25	0	0.0	**5**	**5.0**
NSC309401	24198955	12.5	6.25	100	100	6.25	1	0.5	**3**	**3.5**
Streptovaricin C	135431273	6.25	6.25	25	12.5	6.25	0	0.5	**2**	1.5
NSC204262	5216088	25	25	12.5	25	12.5	0	0.5	1	0.5
NSC654260	375121	100	12.5	100	50	12.5	**3**	**2.0**	0	1.0
Chaetochromin	53277	25	25	50	25	25	0	0.5	**1**	**0.5**
NSC53275	9568176	100	50	50	25	25	1	1.0	1	1
Celastrol	122724	200	50	200	50	50	**2**	**2.0**	0	0.0

^*a*^ $L2_{\left(UM\pm Cef\right)}=log_{2}\left(\frac{MIC_{UM-Cef}}{MIC_{UM+Cef}}\right).$

^*b*^ $AL2_{\left(\pm Cef\right)}\;=Avg\left[log_{2}\left(\frac{MIC_{UM-Cef}}{MIC_{UM+Cef}}\right),\;log_{2}\left(\frac{MIC_{PM-Cef}}{MIC_{PM+Cef}}\right)\right].$

^*c*^ $\;L2_{\left(UM/PM-Cef\right)}=log_{2}\left(\frac{MIC_{UM-Cef}}{MIC_{PM-Cef}}\right).$

^*d*^ $AL2_{\left(UM/PM\right)}\;=Avg\left[log_{2}\left(\frac{MIC_{UM-Cef}}{MIC_{PM-Cef}}\right),\;log_{2}\left(\frac{MIC_{UM+Cef}}{MIC_{PM+Cef}}\right)\right].$

^*e*^ −Cef = in the absence of cefoxitin; +Cef = in the presence of 8 mg/L cefoxitin; boldface numbers indicate significant values.

^*f*^ UM, un-metabolized.

^*g*^ PM, post-metabolized.

^*h*^ CID, compound identifier.

^*i*^ 4-QDA, 4-quinazolinediamine.

Several of the identified agents (Table 1) are previously known antibacterial agents. Clorobiocin is an aminocoumarin DNA gyrase inhibitor similar to novobiocin (17, 18). Ethyl violet is a homolog of crystal (methyl) violet that is a well-known antibacterial agent of unknown mechanism (19). Hitachimycin (stubomycin) is a generally cytotoxic agent with Gram-positive antibacterial activity isolated from streptomyces cultures (20, 21). Streptovaricin C is a known antibiotic that inhibits mRNA polymerase (22). Several other agents on this list have been identified as having anti-MRSA activity in publicly available library screening databases [ChEMBL CHEMBL4296184 (23) and PubChem assay identifiers (AIDs) 1259311 and 1409573].

### Comparative MIC analyses to identify agents synergistic with cefoxitin

Comparisons between MIC values are included in Table 1 to highlight the effect of added cefoxitin on compound MICs and the effect of microsomal metabolism on MICs. The L2_(±Cef)_ values represent simple comparisons between UM compound MICs in the absence and presence of cefoxitin:

L2_(±Cef)_ = log_2_ $\left(\frac{{MIC}_{UM-Cef}}{{MIC}_{UM+Cef}}\right)$ .

This represents the log_2_-fold change for the UM − Cef/UM + Cef MIC ratio. An L2 for UM − Cef vs PM − Cef can be defined similarly (L2_(UM/PM)_), which reflects the change in between the UM − Cef and PM − Cef MIC ratio. The AL2 values represent the average effect of added cefoxitin on UM and PM MIC values or of compound metabolism on both −Cef and +Cef values, as presented previously (12) and as defined in the footnote of Table 1. Parameter values ≥ 2 (fourfold changes, highlighted in Table 1) indicate significantly increased potency (lower MIC), and values ≤−2 (highlighted in Table 1) indicate significantly decreased potency (higher MIC). Five compounds demonstrated L2_(±Cef)_ ≥ 2 values, identifying these as likely synergistic agent combinations with cefoxitin, and worthy of follow-up checkerboard analyses. No compounds demonstrated L2_(UM/PM)_ ≥ 2 values indicative of a substantially more active metabolite, and no further follow-up on active metabolite identification was therefore performed.

### Checkerboard analyses

**Fig 1:**
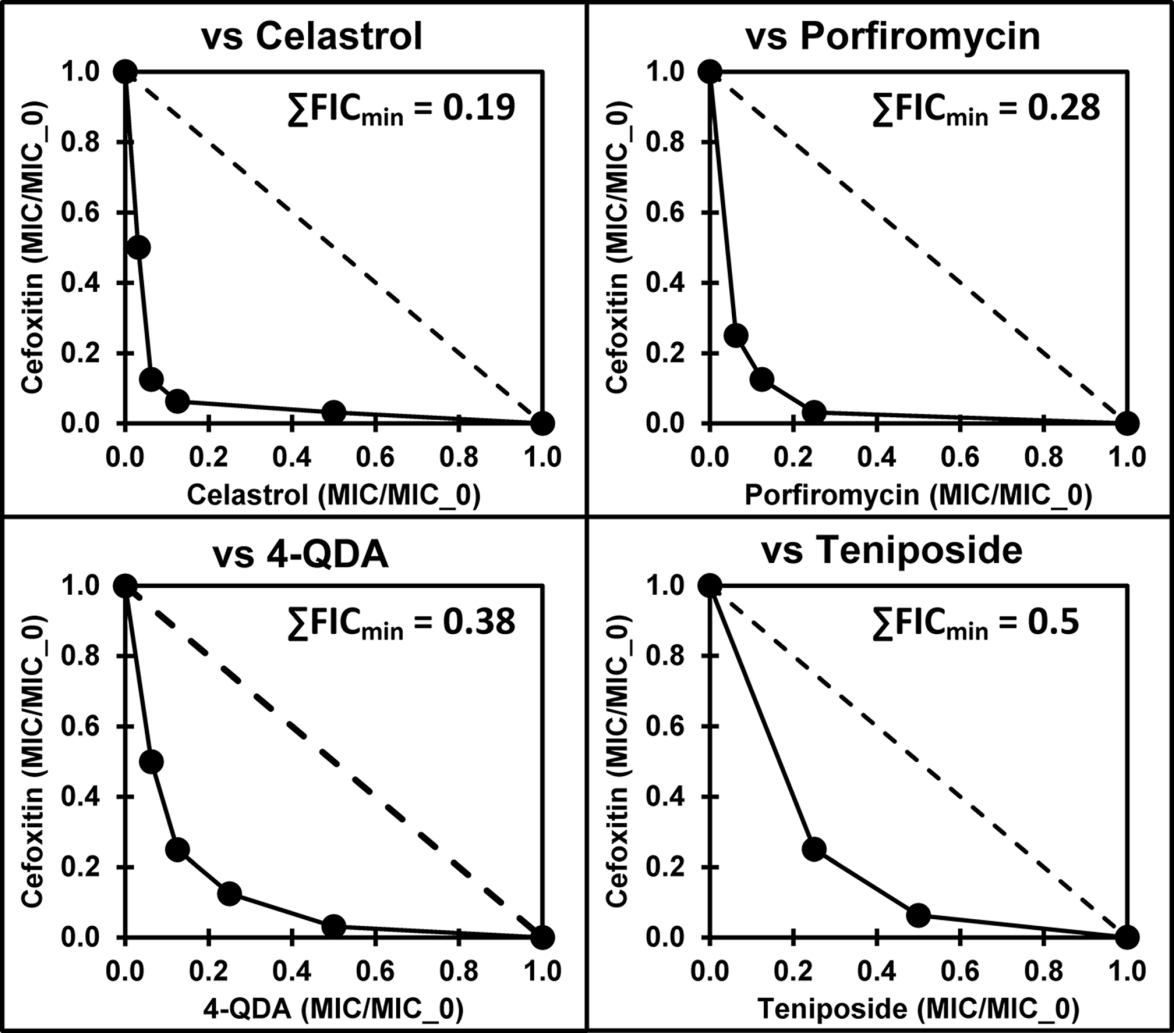
Checkerboard assay results as isobolograms for combinations of cefoxitin with celastrol, porfiromycin, 4-quinazolinediamine (4-QDA), and teniposide against MRSA (ATCC 43300). The dashed line in the isobolograms is for the no interaction (additive MICs) curve. MICs for other agents alone are given in Table 1.

Five compounds in Table 1 showed apparent significant synergy (L2_(±Cef)_ ≥ 2). Follow-up checkerboard assays were performed for all these except NSC654260, which was not available in sufficient amounts for this analysis. This confirmed the synergy of cefoxitin with all four of the tested L2_(±Cef)_ ≥ 2 compounds (Fig. 1), ranging from relatively strong synergy (∑FIC_min_ = 0.19) for celastrol to relatively weak synergy (∑FIC_min_ = 0.5) for teniposide. There does not appear to be a common mechanistic relationship between these four synergistic-with-cefoxitin compounds.

### Identification and confirmation of folate/thymidine biosynthesis inhibitors

**Fig 2:**
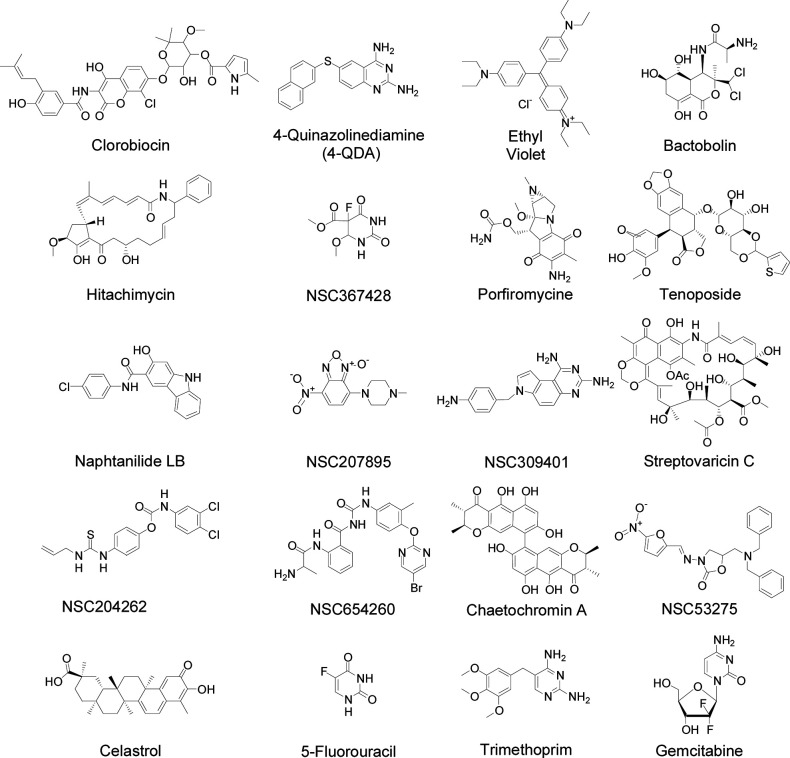
Structures of active or referenced compounds.

Two of the compounds in Table 1 had the diaminopyrimidine pharmacophore associated with folate reductase inhibitors such as trimethoprim [4-quinazolinediamine (4-QDA) and NSC309401, Fig. 2]. Folate is required for the synthesis of thymidine, and the addition of thymidine can be used to reverse the action of folate/thymidine biosynthesis inhibitors (15). It was therefore expected that redetermining the MICs of the compounds in Table 1 in the absence and presence of 4-µM (1-µg mL^−1^) thymidine (±Thy) could be used to identify folate/thymidine biosynthesis inhibitors within this group (Table 2). This identified three compounds with significantly increased MICs in the presence of thymidine (L2_(±Thy)_ ≥ 2): the two diaminopyrimidine compounds (4-QDA and NSC309401) as well as the fluorine substituted pyrimidine analog NSC367428 (Table 2).

**TABLE 2:** Additional compound information^*a*^ ^,^ ^*d*^

Name	Thy	+Thy	L2_(±Thy)_^s^	MBC/MIC^*e*^ (−Thy)	C/S^*b*^	PubChem NSC	PubChem CID^*f*^	PubChem AID^*b*^	Toxic^*c*^
Clorobiocin	0.049	0.049	0	4	C	227186	54677920	1409572	Y
4-QDA^*g*^	0.78	100	**7**	>8	S	305780	16682542	720589	Y
Ethyl violet	3.1	6.25	1	2	C	8675	16955	1409572	Y
Bactobolin	3.1	6.25	1	>8	S	325014	54676871	1409572	Y
Hitachimycin	1.6	1.6	1	2	C	343256	54598584	1409572	Y
NSC367428	3.1	100	**5**	4	C	367428	339703	1409572	N
Porfiromycin	6.25	6.25	1	2	C	56410	244989	1409572	Y
Teniposide	25	25	0	4	C	122819	54610154		Unk
Naphtanilide LB	12.5	12.5	0	>8	S	50651	67238	504648	N
NSC207895	6.25	12.5	1	4	C	207895	42640	1409572	Y
NSC309401	12.5	100	**3**	4	C	309401	24198955	1409572	Y
Streptovaricin C	3.1	6.25	1	2	C	19990	135431273		Unk
NSC204262	100	100	0	2	C	204262	5216088	504648	N
NSC654260	100	100	0	1	C	654260	375121	504648	N
Chaetochromin	25	25	0	1	C	345647	53277	1409572	Y
NSC53275	100	100	0	1	C	53275	9568176		Unk
Celastrol	100	50	1	2	C	70931	122724	1409572	Y

^*a*^ C = −cidal; S = −static.

^*b*^ PubChem bioassay [assay identifier (AID) record] results for cytotoxicity testing. The AID504648 screen used A549 ARE_Flux cells, the AID720589 screen used HepG2 cells, and the AID1409572 screen used HEK293 cells.

^*c*^ $\;L2_{\left(\pm Thy\right)}\;=log_{2}\left(\frac{MIC_{+Thy}}{MIC_{-Thy}}\right).$

^*d*^ MICs (µM) for the top NCI diversity set V compounds against MRSA (ATCC #43300) in the absence (−Thy) and presence (+Thy) of 4-µM of thymidine. The associated L2 values identify those agents targeting folate/thymidine biosynthesis (bold entries). MBC/MIC ratios and −static vs −cidal designations (C/S column, −Thy) are included in the MBC/MIC column. The toxic column summarizes toxicity results from other screening efforts identified in the indicated PubChem bioassay (AID) records.

^*e*^ MBC/MIC, minimum bactericidal concentration/minimum inhibitory concentration.

^*f*^ CID, compound identifier.

^*g*^ 4-QDA, 4-quinazolinediamine.

**Fig 3:**
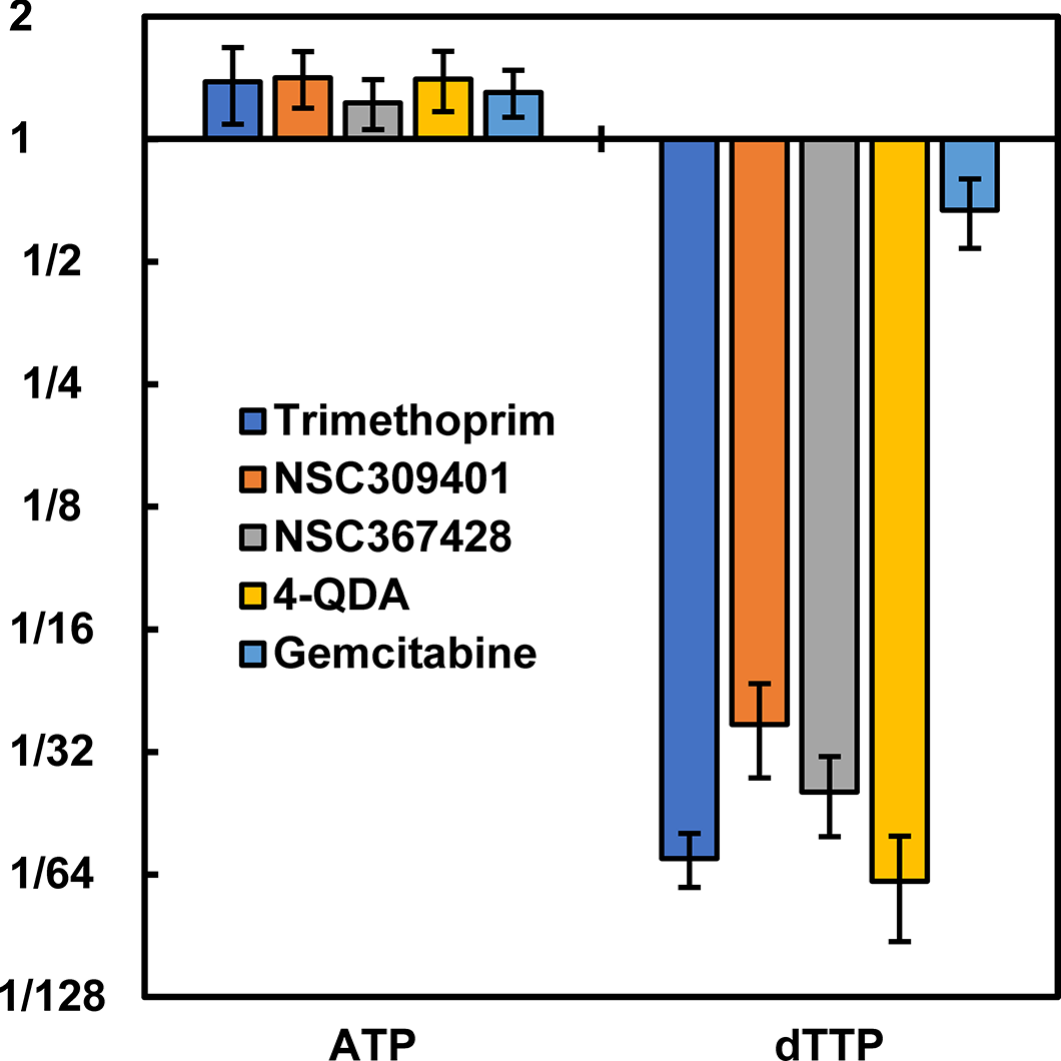
Fold changes in the levels of ATP and dTTP upon exposure to 4× MIC of different agents for 15 min relative to an untreated control.

To further confirm these as thymidine biosynthesis inhibitors, an ion-pairing LC-MS/MS method was developed for dTTP, with ATP as a control nucleotide (Table 3) similar to the method developed for the UDP-linked intermediates in the bacterial peptidoglycan biosynthesis pathway (24). This method was used to determine the level of dTTP after MRSA exposure to the putative folate/thymidine biosynthesis inhibitors, with trimethoprim included as a positive control and gemcitabine (12) included as a negative control. These LC-MS/MS results (Fig. 3) clearly demonstrate substantial dTTP level suppression for NSC309401, NSC367428, and 4-QDA. The two diaminopyrimidine-containing agents (4-QDA and NSC309401) are likely folate reductase inhibitors. The mechanism of thymidine biosynthesis inhibition by the fluoropyrimidine NSC367428 is unknown, but it is structurally similar to 5-fluorouracil (Fig. 2). This ±thymidine approach to the quick identification of folate/thymidine biosynthesis inhibitors is a simple extension to the general synergy screening approach used in this and several prior studies. Since folate biosynthesis is an essential bacterial biochemical pathway, this approach could be expanded for the large-scale identification of novel agents targeting this essential and druggable pathway.

**TABLE 3:** Retention time (*t*_*R*_) and MS/MS parameters for ATP and dTTP quantification^*a*^

	*t*_*R*_ (min)	Q1	Q3	DP (V)	EP (V)	CE (V)
ATP	12.6	506.0	158.9	-60	-3	-44
dTTP	14.3	481.0	158.9	-55	-4	-40

^*a*^ Global method parameters were TEM (source temperature), 300°C; IS (ion spray voltage), −4,500 V; GS1 and GS2 (gas flows), 50 (arbitrary units); CAD gas, medium.

### Toxicity data

Most of the identified compounds have been previously screened for cytotoxicity, and PubChem NSC identifiers, compound identifiers (CIDs) , and cytotoxicity (bioassay) AIDs and screening results are also included in Table 2.

### Spectrum of activity

To further assess the potential of this group of NCI compounds as anti-MRSA and antibacterial agents, spectrum of activity was determined against several MRSA strains, one strain of vancomycin-resistant *Enterococcus* (VRE) *faecium*, one strain of VRE *faecalis*, and one strain of *Escherichia coli* (Table 3). Only NSC367428, the fluoropyrimidine derivative (Fig. 2), demonstrated appreciable activity against *E. coli*. This is in contrast to the structurally similar 5-fluorouracil, which did not show activity against *E. coli* (12). Clorobiocin showed the best MRSA spectrum of activity, followed by 4-QDA, bactobolin, streptovaricin C, ethyl violet, NSC367428, and hitachimycin, based on average MRSA MIC. Naphtanilide LB and, to a lesser degree clorobiocin were unusual in their selectivity to certain MRSA strains.

### Conclusions

A library screening effort was performed with the NCI diversity set V against MRSA to both identify novel antibacterial metabolites and synergistic agents with cefoxitin. In contrast to a prior similar screen of an FDA-approved drug library against MRSA (12), human microsome metabolism of the NCI library did not result in the identification of any new active metabolites. However, similar to this prior FDA screen, screening the NCI library in the absence and presence of cefoxitin allowed for the identification of several synergistic combinations with cefoxitin: celastrol, porfiromycin, 4-QDA, and tenoposide. Two of these synergistic agents, celastrol and porfiromycin, are DNA-damaging agents (25, 26), teniposide is a DNA gyrase inhibitor (27), and 4-QDA is a folate/thymidine biosynthesis inhibitor as demonstrated in this study. There does not seem to be an obvious common mechanistic basis for the synergy of these four agents with cefoxitin. The identification of several folate/thymidine biosynthesis inhibitors using a ±thymidine counter screen-identified three compounds (4-QDA, NSC367428, and NSC309401) as folate/thymidine biosynthesis inhibitors, and these were confirmed as able to suppress dTTP biosynthesis in MRSA by LC-MS. 4-QDA may provide a lead for further folate biosynthesis inhibitors. Of the top-ranked hits, all appear bactericidal except 4-QDA, bactobolin, and naphthnilide LB. Several other agents identified in this screen are unknown but potentially interesting mechanisms including naphtanilide LB, NSC207895, NSC204262, NSC654260, and NSC53275. Of these, only NSC207895 has been identified as cytotoxic to human cells in prior screening efforts (Table 2). Further mechanistic studies of these agents may provide new targets for focused drug discovery and refinement efforts.

## MATERIALS AND METHODS

### General

The NCI diversity set V library of 1,593 compounds was from the Division of Cancer Treatment and Diagnosis (DCTD) of the NCI. All other materials were as described previously (12).

### Library replication, addition of metabolism, and antibacterial control compounds

The NCI diversity set V was delivered in 96 well plates in Columns 2–11, 20 plates total, with each well containing 20 µL of a 10-mM solution of a compound in DMSO. Antibiotic controls (20 µL of 10 mM stock solutions of vancomycin, fosfomycin, ampicillin, doxycycline, or chloramphenicol) were added to Column 1 of each library plate. Microsomal (CYP) substrate controls (20 µL of 10 mM stock solutions of phenacetin, tolbutamide, dextromethorphan, coumarin, chlorzoxazone, or diclofenac) were added to Column 12 of each library plate. Aliquots (10 µL) of library samples were transferred to 96-well plates using a liquid-handling workstation (Biomek 3000) and diluted with 90 µL DMSO to provide UM working plates at 1 mM.

### *In vitro* microsomal metabolism to provide the PM library

For PM library preparation, the remaining 10 µL of each sample in DMSO was dried by freezing the plates at –80°C and drying under a strong vacuum (<50 µmHg) in a Genevac Quatro centrifugal concentrator (DMSO can interfere with microsomal metabolism reactions). The dried library plates were metabolized with human liver microsomes as described previously (12). To each well was added 10 µL acetonitrile/water (20%/80%, vol/vol) to redissolve samples. The plates were incubated for 2 h at 35°C, followed by the addition of 490 µL of freshly prepared (on ice) microsomal reaction mixture containing 50 mM potassium phosphate pH 7.4, 3 mM MgCl_2_, 5 mM glucose-6-phosphate, 1 unit mL^−1^ glucose-6-phosphate dehydrogenase, 1 mM NADP^+^, and 0.5 mg mL^−1^ total microsomal protein. Reaction mixtures were incubated for 24 h at 35°C with gentle rocking. Library plates were then centrifuged at 4,000 *g* for 30 min at 4°C, and 400 µL of the supernatants was then transferred to sterile 96 well plates. To the residues was added 100 µL DMSO, and the samples were mixed thoroughly. Library plates were centrifuged again at 4,000 *g* for 30 min, and 150 µL of the supernatants was removed and combined with the first extracts. The resulting extracts were frozen at –80°C and dried under strong vacuum (<50 µmHg) in a Genevac Quatro centrifugal concentrator. These PM library samples were then reconstituted in 100-µL DMSO to provide a 1-mM PM NCI working library. Both UM and PM working libraries were stored in U-bottom polypropylene storage plates at –80°C. Samples of wells containing microsomally metabolized drug controls from PM plates were analyzed by LC-MS/MS to provide a relative measure of metabolism. The percent metabolism of these control drugs was 52%, 55%, 60%, 66%, 95%, and 100% for tolbutamide, dextromethorphan, chlorzoxazone, phenacetin, diclofenac, and coumarin respectively. These controls demonstrate that the metabolism conditions employed in this study were sufficient to achieve a relatively high degree of metabolism.

### UM/PM vs ±Cef library screen against MRSA

Four sets of library screens were performed (UM − Cef, UM + Cef, PM − Cef, and PM + Cef), as described previously for an FDA-approved drug library screen (12), with the modification that 2 µL of library samples at 1 mM was used. During the bacterial incubation step, this provided 100 µM compound concentrations, rather than 200 µM as in the previously described study (12). Plates were frozen at –80°C and dried as described above. To each well in each set was added 20 µL cation-adjusted Mueller-Hinton (CAMH) broth containing 4,000 CFU MRSA (ATCC 43300) and containing either no cefoxitin for –Cef screens or +8 µg mL^−1^ cefoxitin (equal to 1/4× MIC) for +Cef screens. Plates were incubated for 48 h at 35°C. Fresh CAMH broth (10 µL) was then added to the wells of these four sets of plates, followed by incubation for 2 h at 35°C, to restart active cell growth. To the wells of these plates was then added 6 µL of 100 µg mL^−1^ resazurin (sodium salt) (28 – 30). The plates were incubated for another 2 h at 35°C, and the 570/600 fluorescence ratio was measured in a Molecular Devices SpectraMax M5 multimode microplate reader. The resulting data were processed and analyzed using MATLAB scripts (The Mathworks, Natick, MA, USA) to identify active wells using a cutoff value between known actives (antibiotic controls) and known inactives (microsomal controls). A merged hit list was generated, in which a compound was included in the merged hit list if it demonstrated activity under any of the four test conditions (UM − Cef, UM + Cef, PM − Cef, or PM + Cef).

### Hit picking and MIC determination

Follow-up MIC determinations for identified hits were performed as described in detail previously (12) using a resazurin (Alamar Blue)-based colorimetric assay (28 – 30). This assay gives comparable results to standard clinical MIC methods for *S. aureus* with increased sensitivity in a 384-well plate format. MICs were determined for all actives by hit picking 2 µL samples from both UM and PM working plates (two sets from each) into the first columns of 384 well plates (four sets total, for UM – Cef, UM + Cef, PM – Cef, and PM + Cef MIC determinations). These samples were then serially diluted in steps of two across the plates with DMSO using an Integra Viaflo Assist-automated multichannel pipette. The last column was left blank (DMSO only). These plates were frozen at –80°C and dried under a strong vacuum as described above. To each well in each set was added 20-µL CAMH broth containing 4,000 CFU MRSA (ATCC 43300) and containing either no cefoxitin for –Cef MICs or 8 µg mL^−1^ Cef for +Cef MICs. This provided MIC plates with 100 µM as the highest test agent concentration. Incubation and resazurin treatment were as described above. MICs were determined using a cutoff midway between known active and inactive samples. All MICs were determined at least in triplicate.

### Minimum bactericidal concentrations

Minimum bactericidal concentrations (MBCs) were determined by preparing and drying UM − Cef MIC plates as described above. To each well was added 20 µL CAMH broth containing 8,000 CFU MRSA, and the plates were incubated for 18–24 h. After resazurin addition and development as described above, 20 µL from the 1× MIC, 2× MIC, and 4× MIC wells for each compound was removed and plated on CAMH agar plates. The plates were incubated overnight, and colony counts were assessed. A greater than 1,000-fold decrease from anticipated colony counts was scored as bactericidal, and a less than 1,000-fold decrease as bacteriostatic. All MBCs were determined at least in triplicate and reported as the ratio of the MBC to the MIC (Table 2).

### Checkerboard assays to confirm synergy with cefoxitin

Several agents showed lower MICs in the presence of cefoxitin (Table 1), indicative of potential synergistic activity. Checkerboard assays (31) were used to confirm and assess synergy for 4-QDA, celastrol, teniposide, streptovaricin, porfiromycin, and ethyl violet with cefoxitin, as described previously (12). All checkerboard assays were performed in triplicate. Data were plotted as isobolograms and reported as the minimum sum of fractional inhibitory concentrations (∑FIC_min_ values in Fig. 1, also referred to as FICI values) (32).

### ±Thymidine counter screen and LC-MS/MS confirmation for folate/thymidine biosynthesis inhibitors

The effects of folate/thymidine biosynthesis inhibitors on MRSA can be reversed by the addition of thymidine to the culture media (15). This effect was therefore used to assess Table 1 by redetermining the UM − Cef MICs in the absence and presence of 4 µM (1-µg mL^−1^) thymidine (Table 2). This identified three agents with significant L2_(±Thy)_ values.

To further confirm these three agents as thymidine biosynthesis inhibitors, an ion-pairing LC-MS/MS assay was developed for ATP and dTTP using the same approach as previously described for UDP-linked intermediates in the bacterial cell wall biosynthesis pathway (24) (Table 3). Antibiotic-treated bacterial cultures were prepared as described in detail previously (24). MRSA cultures were grown in CAMH media to the mid-log phase (OD_600_ = 0.5), and 50 mL of this mid-log phase was transferred to baffled 250 mL culture flasks and treated with the test agent at 4× MIC (Table 2, Thy values) for 15 min. The tested agents were NSC367428, 4-QDA, and NSC309401, with trimethoprim included as a positive control and gemcitabine included as a negative control. A non-antibiotic control flask was also included. The flasks were incubated at 35°C with shaking for 15 min. Flasks were then rapidly chilled in an ice slush bath, and the samples from individual flasks were collected in quadruplicate and stored on ice for up to 15 min prior to centrifugations and processing for metabolite extraction, as described above. Samples were analyzed for ATP and dTTP using the LC-MS/MS parameters described in Table 3. The results from this experiment are reported in Fig. 2.

### Spectrum of activity

MICs were determined for many of the Table 1 (UM − Cef) agents against a panel of bacterial strains to assess the spectrum of activity (Table 4). The strains tested were MRSA strains F-182 (ATCC 43300), N315 (BEI NR-45898), HI022 (BEI NR-30550), MN8 (BEI NR-45918), TCH70 (BEI HM-139), RN1 (BEI NR-45904), COL (BEI NR-45906), U9N0, one strain of VRE *faecium* (clinical), one strain of VRE *faecalis* (ATCC 2365), and one strain of *E. coli* K12 (BEI MG1655).

**TABLE 4:** Spectrum of activity of NCI compounds (MIC, µM)

Compound *^a^*	MRSA^*e*^ (F-182) *^b^*	MRSA (N315)	MRSA (HI022)	MRSA (MN8)	MRSA (TCH70)	MRSA (RN1)	MRSA (COL)	MRSA (U9N0)	VRE^*g*^ (*faecium*, clinical)	VRE (*faecalis*, 2365)	*E. coli* (K12)
Clorobiocin	0.10	0.10	0.10	NA^*c*^	0.20	0.20	0.024	NA^*c*^	6.25	25	NA^*c*^
4-QDA^*f*^	0.39	3.1	1.6	0.78	1.6	1.6	6.25	3.1	1.6	NA^*c*^	50
Ethyl violet	1.6	6.25	3.1	3.1	6.25	6.25	6.25	6.25	6.25	12.5	50
Bactobolin	3.1	3.1	3.1	3.1	3.1	3.1	3.1	6.25	12.5	NA^*c*^	50
Hitachimycin	1.6	0.8	12.5	1.6	12.5	3.1	3.1	12.5	1.6	6.25	NA^*c*^
Porfiromycin	12.5	12.5	12.5	12.5	12.5	12.5	12.5	NA	12.5	12.5	
Streptovaricin C	6.25	3.1	6.25	3.1	1.6	3.1	1.6	6.25	25	50	NA^*c*^
Naphtanilide LB	6.25	1.6	NA^*c*^	NA^*c*^	NA^*c*^	12.5	NA^*c*^	NA^*c*^	NA^*c*^	NA^*c*^	NA^*c*^
NSC207895	12.5	3.1	12.5	50	12.5	12.5	12.5	25	12.5	12.5	NA^*c*^
NSC309401	12.5	25	50	12.5	25	12.5	50	12.5	NA^*c*^	NA^*c*^	6.25
NSC367428	3.1	12.5	6.25	6.25	6.25	6.25	1.56	3.1	NA^*c*^	NA^*c*^	NA^*c*^
Trimethoprim *^d^*	25	25	12.5	12.5	12.5	25	12.5	12.5	1.6	NA^*c*^	3.1
Vancomycin *^d^*	1.6	0.39	1.6	0.39	0.78	0.78	0.78	1.6	NA	NA^*c*^	NA^*c*^
Doxycycline *^d^*	NA^*c*^	NA^*c*^	NA^*c*^	NA^*c*^	NA^*c*^	NA^*c*^	NA^*c*^	NA^*c*^	16	3.1	NA^*c*^

^*a*^ Structures are shown in the figure below.

^*b*^ ATCC 43300 MRSA strain used for library screening. Other vendor IDs are given in the text.

^*c*^ Not active (NA) at 50 µM, the highest concentration used in these MIC determinations.

^*d*^ Control antibiotic.

^*e*^ MRSA, methicillin-resistant *Staphylococcus aureus*.

^*f*^ 4-QDA, 4-quinazolinediamine.

^*g*^ VRE, vancomycin-resistant *Enterococcus*.

## SUPPLEMENTAL MATERIAL

Supplemental material spectrum.00541-23-s0001.pdf Tables S1 and S2.

ASM does not own the copyrights to Supplemental Material that may be linked to, or accessed through, an article. The authors have granted ASM a non-exclusive, world-wide license to publish the Supplemental Material files. Please contact the corresponding author directly for reuse.
