# Supplementary material for: Screening the NCI diversity set V for anti-MRSA activity: cefoxitin synergy and LC-MS/MS confirmation of folate/thymidine biosynthesis inhibition
Source: Microbiol Spectr. 2023 Oct 27;11(6):e00541-23. doi: 10.1128/spectrum.00541-23 (PMC10715016; doi:10.1128/spectrum.00541-23)
Supplement: Supplemental material — Tables S1 and S2. [file spectrum.00541-23-s0001.pdf]

Supplementary Information

"Dimensionally Enhanced Screening of the NCI Diversity Set V for Anti-MRSA Activity and Cefoxitin Synergy"

Shivani Gargvanshi, Gioia Heravi, Navid J. Ayon, and William G. Gutheil*

Division of Pharmacology and Pharmaceutical Sciences, School of Pharmacy, University of Missouri-Kansas City, 2464 Charlotte Street, Kansas City, MO 64108

*Corresponding author: William G. Gutheil, Division of Pharmacology and Pharmaceutical Sciences, School of Pharmacy, University of Missouri Kansas City, 2464 Charlotte Street., Kansas City, MO, 64108, USA, Tel.: (816) 235-2424; Fax: (816) 235-5779; E- mail: gutheilw@umkc.edu

Running title: Dimensionally enhanced screening against MRSA.

Keywords: Library screening; Drug repurposing; Staphylococcus aureus; Microsome; P450; Metabolism; Chemical Diversity; Synergy; Antibiotic drug resistance; MRSA.

Contents

Table S1. List of actives (validated MIC≤100 μM) from library screening against MRSA (ATCC 43300) (UM/PM vs –/+ 8 μg mL^{-1}) ranked by lowest minimum MIC.

Table S2. List of inactive compounds (MIC>100 μM) from library screening against MRSA (ATCC 43300).

Table S1. List of active compounds (validated MIC≤100 μM) from NCI Diversity Set V library screening against MRSA (ATCC 43300) (UM/PM vs –/+ 8 μg mL^{-1} Cefoxitin) ranked by lowest minimum MIC.

		UM MICs (μM)		PM MICs (μM)			
Name/NSC_No	PubChem_CID	–Cef	+Cef	–Cef	+Cef	Min_MIC	L2 (-/+Cef)
Clorobiocin	54677920	0.10	0.10	0.10	0.049	0.049	0
4-QDA	16682542	0.39	0.10	25	25	0.10	2
Ethyl Violet	16955	1.6	0.78	25	12.5	0.78	1
Bactobolin	54676871	3.1	1.6	12.5	6.25	1.6	1
Hitachimycin	54598584	1.6	1.6	3.1	3.1	1.6	0
NSC367428	339703	3.1	3.1	50	25	3.1	0
Porfiromycin	244989	12.5	3.1	25	25	3.1	2
Teniposide	54610154	12.5	3.1	25	12.5	3.1	2
Naphtanilide LB	67238	6.25	6.25	25	12.5	6.25	0
NSC207895	42640	6.25	6.25	200	200	6.25	0
NSC309401	24198955	12.5	6.25	100	100	6.25	1
Streptovaricin C	135431273	6.25	6.25	25	12.5	6.25	0
NSC204262	5216088	25	25	12.5	25	12.5	0
NSC654260	375121	100	12.5	100	50	12.5	3
Chaetochromin	53277	25	25	50	25	25	0
NSC53275	9568176	100	50	50	25	25	1
Celastrol	122724	200	50	200	50	50	2
NSC204262	5216088	100	100	12.5	25	12.5	
Ellipticine	3213	25	25	25	25	25	
NSC344494	6512428	200	50	200	200	50	
CDDO-Im	9958995	200	50	200	200	50	
CID 319089	319089	50	50	200	100	50	
NSC38090	236065	100	50	100	100	50	
Niazo	96213	200	50	200	200	50	
NSC11667	240350	50	50	100	100	50	
NSC11667	223752	200	200	100	50	50	
NSC11668	54600468	50	50	100	100	50	
NSC11668	223753	200	200	100	50	50	
NSC177407	67275	50	200	200	200	50	
NSC177407	5383615	100	100	100	50	50	
NSC332670	56909	50	50	100	100	50	
NSC332670	332972	200	200	200	50	50	
NSC341196	328773	50	50	200	200	50	
NSC341196	334739	200	200	100	50	50	
NSC369066	339983	100	50	50	50	50	
NSC522131	313619	50	100	200	200	50	
NSC522131	351549	100	100	100	50	50	
Albacarcin V	122815	100	100	100	100	100	
NSC149286	5382674	200	100	200	200	100	
NSC186200	5842286	200	100	200	100	100	
NSC369070	135493774	100	200	200	200	100	
NSC137399	6509134	200	200	200	100	100	
NSC33353	40492789	200	100	100	100	100	

NSC329249	332429	200	200	200	100	100
NSC33005	95746	200	100	200	200	100
NSC317003	13504751	100	100	100	100	100
NSC133071	280859	200	100	200	200	100
NSC159566	293227	200	100	200	100	100
NSC147358	287384	200	100	200	200	100
NSC177365	5351256	100	200	200	200	100
NSC215721	3967840	200	200	100	200	100
Naphthol AS-OL	67274	200	200	200	100	100
NSC138389	283529	200	100	200	200	100
NSC13156	224574	100	200	100	100	100
NSC30260	232590	100	100	200	200	100
NSC407628	82011	200	100	200	200	100
NSC622689	360560	100	100	200	200	100
NSC311727	100520	100	100	200	200	100
NSC133114	280895	100	200	200	200	100
Benzbromarone	2333	200	200	200	100	100
NSC116339	5381366	200	100	200	200	100
Daunomycin 3-oxime HCl	54606703	200	100	200	200	100
Malonoben	5614	200	200	200	100	100
Mequitazine	4066	200	100	200	200	100
Methiothepin maleate	5358812	200	100	200	200	100
Methyl Streptonigrin	18834	200	200	200	100	100
NSC601359	353380	100	200	200	100	100
Enpiroline	328144	100	100	200	200	100
NSC106208	97205	200	100	200	200	100
NSC302584	163121	200	100	200	200	100
Redoxal	72571	100	100	200	100	100
Sulfaquinoxaline	5338	100	100	100	100	100
Tricinolone acetophenonide	235856	200	100	200	200	100
NSC607097	122737	100	100	200	200	100
Vacquinol-1	224644	200	200	200	100	100
Wander	65558	100	100	200	200	100

Table S2. List of inactive compounds (MIC>100 μM) from library screening against MRSA (ATCC 43300).

Compound	CAS Number
Octopamine	770-05-8
10-Deacetylbaccatin-III	32981-86-5
1-Hexadecanol	36653-82-4
2-Methoxyestradiol	362-07-2
2-Thiouracil	141-90-2
5-Aminolevulinic	106-60-5
9-Aminoacridine	90-45-9
Abitrexate	59-05-2
Acadesine	2627-69-2
Acarbose	56180-94-0
Acebutolol	34381-68-5
Aceclidine	6109-70-2
Acemetacin	53164-05-9
Acetanilide	103-84-4
Acetarsone	97-44-9
Acetylcholine	60-31-1
Acetylcysteine	616-91-1
Acipimox	51037-30-0
Acitretin	55079-83-9
Aclidinium	320345-99-1
Acyclovir	59277-89-3
Adapalene	106685-40-9
Adefovir	142340-99-6
Adenine	2922-28-3
Adenosine	58-61-7
Adiphenine	50-42-0
Adrenalone	62-13-5
Afatinib	439081-18-2
Agomelatine	138112-76-2
Albendazole	54965-21-8
Albendazole	54029-12-8
Alfacalcidol	41294-56-8
Alfuzosin	81403-68-1
Alibendol	26750-81-2
Aliskiren	173334-58-2
Allopurinol	315-30-0
Allylthiourea	109-57-9
Almotriptan	181183-52-8
Alprostadil	745-65-3
Altrenogest	850-52-2
Altretamine	645-05-6
Alverine	5560-59-8
Amantadine	665-66-7
Ambrisentan	177036-94-1
Amfebutamone	31677-93-7
Amfenac	61618-27-7
Amidopyrine	58-15-1
Amiloride	2016-88-8
Amiloride	17440-83-4
Aminocaproic	60-32-2
Aminoglutethimide	125-84-8
Aminophylline	317-34-0
Aminosalicylate	133-10-8
Aminothiazole	96-50-4
Amiodarone	19774-82-4
Amisulpride	71675-85-9
Amitriptyline	549-18-8
Amlodipine	88150-42-9
Amlodipine	111470-99-6
Glycyrrhizinate	1407-03-0
Amorolfine	78613-38-4
Amphotericin	1397-89-3
Ampicillin	7177-48-2
Ampiroxicam	99464-64-9
Amprenavir	161814-49-9
Amprolium	137-88-2
Anagrelide	58579-51-4
Anastrozole	120511-73-1
Aniracetam	72432-10-1
Anisotropine	80-50-2
Antazoline	2508-72-7
Antipyrine	60-80-0
Apatinib	811803-05-1
Apixaban	503612-47-3
Arbidol	131707-23-8
Arecoline	300-08-3
Argatroban	74863-84-6
Aripiprazole	129722-12-9
Artemether	71963-77-4
Artemisinin	63968-64-9
Articaine	23964-57-0
Asenapine	65576-45-6
Aspartame	22839-47-0
Aspirin	50-78-2
Atazanavir	229975-97-7
Atomoxetine	82248-59-7
Atorvastatin	134523-03-8
Atovaquone	95233-18-4
Atracurium	64228-81-5
Atropine	5908-99-6
Avanafil	330784-47-9
Avobenzone	70356-09-1
Axitinib	319460-85-0
Azacitidine	320-67-2
Azacyclonol	115-46-8
Azaguanine-8	134-58-7
Azaperone	1649-18-9
Azatadine	3978-86-7
Azathioprine	446-86-6
Azelastine	79307-93-0
Azelnidipine	123524-52-7
Azilsartan	147403-03-0
Azilsartan	863031-21-4
Azithromycin	83905-01-5
Azithromycin	117772-70-0
Aztreonam	78110-38-0
Bazedoxifene	198480-56-7
Beclomethasone	4419-39-0
Bemegride	64-65-3
Benazepril	86541-74-4
Bendamustine	3543-75-7
Benidipine	91599-74-5
Benserazide	14919-77-8
Benzbromarone	3562-84-3
Benzocaine	94-09-7
Benzoic	65-85-0
Benzthiazide	91-33-8
Benztropine	132-17-2
Benzydamine	132-69-4
Bephenium	3818-50-6

Bepotastine	190786-44-8
Bergapten	484-20-8
Beta	7235-40-7
Betahistine	5579-84-0
Betamethasone	378-44-9
Betamethasone	5593-20-4
Betamethasone	2152-44-5
Betamipron	3440-28-6
Betapar	1247-42-3
Betaxolol	659-18-7
Betaxolol	63659-19-8
Bethanechol	590-63-6
Bexarotene	153559-49-0
Bextra	181695-72-7
Bezafibrate	41859-67-0
BIBR-1048	211915-06-9
Bicalutamide	90357-06-5
Bimatoprost	155206-00-1
Bindarit	130641-38-2
Biotin	58-85-5
Bisacodyl	30652-11-0
Bisoprolol	104344-23-2
Bleomycin	9041-93-4
Bortezomib	179324-69-7
Bosentan	147536-97-8
Bosutinib	380843-75-4
Brinzolamide	138890-62-7
Bromhexine	611-75-6
Brompheniramine	980-71-2
Broxyquinoline	521-74-4
Brucine	652154-10-4
Budesonide	51333-22-3
Bufexamac	2438-72-4
Buflomedil	35543-24-9
Bumetanide	28395-03-1
Bupivacaine	18010-40-7
Busulfan	55-98-1
Butenafine	101827-46-7
Cabazitaxel	183133-96-2
Calcitriol	32222-06-3
Calcium	17140-60-2
Camylofin	54-30-8
Candesartan	139481-59-7
Captopril	62571-86-2
Carbachol	51-83-2
Carbadox	1791337
Carbamazepine	298-46-4
Carbazochrome	51460-26-5
Carbenicillin	4800-94-6
Carbenoxolone	7421-40-1
Carbidopa	28860-95-9
Carbimazole	22232-54-8
Carfilzomib	868540-17-4
Carprofen	53716-49-7
Carvedilol	72956-09-3
Catharanthine	2468-21-5
Ceftazidime	78439-06-2
Cephalexin	15686-71-2
Cephalomannine	71610-00-9
Cepharanthine	481-49-2
Cetirizine	83881-52-1
Chenodeoxycholic	474-25-9
Chloramphenicol	56-75-7
Chlormezanone	80-77-3
Chlorocresol	59-50-7
Chlorothiazide	58-94-6
Chloroxine	773-76-2
Chlorpheniramine	113-92-8
Chlorpromazine	69-09-0
Chlorpropamide	94-20-2
Chlorprothixene	113-59-7
Chlorquinaldol	72-80-0
Chlorzoxazone	95-25-0
Choline	67-48-1
Chromocarb	4940-39-0
Ciclopirox	29342-05-0
Ciclopirox	41621-49-2
Cilnidipine	132203-70-4
Cilostazol	73963-72-1
Cimetidine	51481-61-9
Cinchophen	132-60-5
Cinepazide	26328-04-1
Cisatracurium	96946-42-8
Cisplatin	15663-27-1
Cladribine	4291-63-8
Clarithromycin	81103-11-9
Clemastine	14976-57-9
Cleviprex	167221-71-8
Climbazole	38083-17-9
Clindamycin	18323-44-9
Clindamycin	21462-39-5
Clindamycin	25507-04-4
Clobetasol	25122-46-7
Clofarabine	123318-82-1
Clofazimine	2030-63-9
Clofibric	882-09-7
Clofoctol	37693-01-9
Clomipramine	17321-77-6
Clonidine	4205-91-8
Clopidogrel	120202-66-6
Clorprenaline	6933-90-0
Clorsulon	60200-06-8
Clozapine	5786-21-0
Cobicistat	1004316-88-4
Conivaptan	168626-94-6
Cortisone	50-04-4
Coumarin	91-64-5
Curcumin	458-37-7
Cyclamic	100-88-9
Cyclandelate	456-59-7
Cyclophosphamide	6055-19-2
Cyclosporine	79217-60-0
Cyproheptadine	969-33-5
Cyromazine	66215-27-8
Cysteamine	156-57-0
Cytidine	65-46-3
Dabrafenib	1195765-45-7
Dacarbazine	891986
Daidzein	486-66-8
Dapoxetine	129938-20-1
DAPT	208255-80-5
Darifenacin	133099-07-7
Darunavir	635728-49-3
Dasatinib	302962-49-8

Decamethonium	541-22-0
Decitabine	2353-33-5
Deferasirox	201530-41-8
Deflazacort	14484-47-0
Dehydroepiandrosterone	53-43-0
Deoxyarbutin	53936-56-4
Deoxycorticosterone	56-47-3
Desloratadine	100643-71-8
Desonide	638-94-8
Detomidine	90038-01-0
Dexamethasone	50-02-2
Dexamethasone	1177-87-3
Dexlansoprazole	138530-94-6
Dexmedetomidine	113775-47-6
Dexmedetomidine	145108-58-3
Dexrazoxane	149003-01-0
Dextrose	50-99-7
Dibenzothiophene	132-65-0
Dibucaine	61-12-1
Diclofenac	15307-79-6
Diclofenac	78213-16-8
Diclofenac	15307-81-0
Dicyclomine	67-92-5
Didanosine	69655-05-6
Dienogest	65928-58-7
Diethylstilbestrol	56-53-1
Difluprednate	23674-86-4
Diltiazem	33286-22-5
Dimethyl	624-49-7
Diminazene	908-54-3
Diperodon	537-12-2
Diphemanil	62-97-5
Diphenhydramine	147-24-0
Diphenylpyraline	132-18-3
Dipyridamole	58-32-2
Dirithromycin	62013-04-1
Disopyramide	22059-60-5
Disulfiram	97-77-8
Divalproex	76584-70-8
DL-Carnitine	461-05-2
D-Mannitol	69-65-8
Docetaxel	114977-28-5
Dofetilide	115256-11-6
Domperidone	57808-66-9
Dopamine	62-31-7
Doripenem	364622-82-2
Doxapram	7081-53-0
Doxazosin	77883-43-3
Doxercalciferol	54573-75-0
Doxofylline	69975-86-6
Doxylamine	562-10-7
Droperidol	548-73-2
Dropropizine	17692-31-8
Drospirenone	67392-87-4
Duloxetine	136434-34-9
Dutasteride	164656-23-9
Dyclonine	536-43-6
Dydrogesterone	152-62-5
Dyphylline	479-18-5
Edaravone	89-25-8
Elvitegravir	697761-98-1
Emtricitabine	143491-57-0

Enalapril	76095-16-4
Enalaprilat	84680-54-6
Enoxacin	74011-58-8
Entacapone	130929-57-6
Entecavir	209216-23-9
Epalrestat	82159-09-9
Epinephrine	51-42-3
Eprosartan	144143-96-4
Erdosteine	84611-23-4
Erlotinib	183319-69-9
Erythromycin	114-07-8
Erythromycin	1264-62-6
Escitalopram	219861-08-2
Esmolol	81161-17-3
Esomeprazole	161973-10-0
Esomeprazole	161796-78-7
Estradiol	50-28-2
Estradiol	313-06-4
Estradiol	979-32-8
Estriol	50-27-1
Estrone	53-16-7
Ethacridine	6402-23-9
Ethambutol	1070-11-7
Ethamsylate	2624-44-4
Ethinyl	57-63-6
Ethionamide	536-33-4
Ethoxzolamide	452-35-7
Ethynodiol	297-76-7
Etodolac	41340-25-4
Etomidate	33125-97-2
Etravirine	269055-15-4
Everolimus	159351-69-6
Evista	82640-04-8
Exemestane	107868-30-4
Famciclovir	104227-87-4
Famotidine	76824-35-6
Famprofazone	22881-35-2
Febuxostat	144060-53-7
Felbamate	25451-15-4
Felodipine	72509-76-3
Fenbendazole	43210-67-9
Fenofibrate	49562-28-9
Fenoprofen	34597-40-5
Fenoprofen	71720-56-4
Fenspiride	5053-08-7
Fenticonazole	73151-29-8
Fesoterodine	286930-03-8
Fexofenadine	153439-40-8
Finasteride	98319-26-7
FK-506	104987-11-3
Flavoxate	3717-88-2
Fluconazole	86386-73-4
Flucytosine	2022-85-7
Fludarabine	21679-14-1
Fludarabine	75607-67-9
Flumazenil	78755-81-4
Flumethasone	2135-17-3
Flunarizine	30484-77-6
Flunixin	42461-84-7
Fluocinolone	67-73-2
Fluocinonide	356-12-7
Fluorometholone	426-13-1

Fluoxetine	56296-78-7
Flurbiprofen	51543-39-6
Flutamide	13311-84-7
Fluticasone	80474-14-2
Fluvastatin	93957-55-2
Fluvoxamine	61718-82-9
Formoterol	43229-80-7
Fosaprepitant	265121-04-8
Fosfomycin	78964-85-9
Fulvestrant	129453-61-8
Furosemide	54-31-9
Gabexate	56974-61-9
Gallamine	65-29-2
Ganciclovir	82410-32-0
Gefitinib	184475-35-2
Gemfibrozil	25812-30-0
Genipin	6902-77-8
Geniposide	24512-63-8
Geniposidic	27741-01-1
Genistein	446-72-0
Gestodene	60282-87-3
Gimeracil	103766-25-2
Ginkgolide	15291-75-5
Glafenine	65513-72-6
Gliclazide	21187-98-4
Glimepiride	93479-97-1
Glipizide	29094-61-9
Gliquidone	33342-05-1
Glyburide	10238-21-8
Guaifenesin	93-14-1
Guanabenz	23256-50-0
Guanidine	50-01-1
Halobetasol	66852-54-8
Haloperidol	52-86-8
Homatropine	51-56-9
Homatropine	80-49-9
Hydrochlorothiazide	58-93-5
Hydrocortisone	50-23-7
Hydroxyurea	127-07-1
Hydroxyzine	2192-20-3
Hyoscyamine	101-31-5
Ibuprofen	15687-27-1
Ibutilide	122647-32-9
Idoxuridine	54-42-2
Iloperidone	133454-47-4
Imatinib	152459-95-5
Imatinib	220127-57-1
Imidapril	89371-37-9
Imipramine	113-52-0
Indapamide	26807-65-8
Indomethacin	53-86-1
Ipratropium	22254-24-6
Irinotecan	97682-44-5
Irinotecan	136572-09-3
Irsogladine	57381-26-7
Isoetharine	7279-75-6
Isoniazid	54-85-3
Isoprenaline	51-30-9
Isosorbide	652-67-5
Isotretinoin	4759-48-2
Isovaleramide	541-46-8
Isoxicam	34552-84-6
Isradipine	75695-93-1
Itraconazole	84625-61-6
Ivabradine	148849-67-6
Ivermectin	70288-86-7
Ketoconazole	65277-42-1
Ketoprofen	22071-15-4
Ketorolac	74103-07-4
Ketotifen	34580-14-8
Lacidipine	103890-78-4
L-Adrenaline	51-43-4
Lafutidine	118288-08-7
Lamivudine	134678-17-4
Lamotrigine	84057-84-1
Lansoprazole	103577-45-3
Lapatinib	231277-92-2
Lapatinib	388082-77-7
Leflunomide	75706-12-6
Lenalidomide	191732-72-6
Letrozole	112809-51-5
Levetiracetam	102767-28-2
Levobetaxolol	116209-55-3
Levonorgestrel	797-63-7
Levosimendan	141505-33-1
Levosulpiride	23672-07-3
Licofelone	156897-06-2
Lidocaine	137-58-6
Linagliptin	668270-12-0
Lincomycin	859-18-7
Liothyronine	55-06-1
Lithocholic	434-13-9
Lomerizine	101477-54-7
Lomustine	13010-47-4
Lonidamine	50264-69-2
Loperamide	34552-83-5
Lopinavir	192725-17-0
Loratadine	79794-75-5
Lornoxicam	70374-39-9
Losartan	124750-99-8
Loteprednol	82034-46-6
Lovastatin	75330-75-5
Loxapine	27833-64-3
L-Thyroxine	51-48-9
Malotilate	59937-28-9
Manidipine	89226-50-6
Maprotiline	10347-81-6
Maraviroc	376348-65-1
Masitinib	790299-79-5
MDV3100	915087-33-1
Mecarbinate	15574-49-9
Meclofenamate	6385-02-0
Medetomidine	86347-15-1
Medroxyprogesterone	71-58-9
Mefenamic	61-68-7
Megestrol	595-33-5
Meglumine	6284-40-8
Melatonin	73-31-4
Meloxicam	71125-38-7
Memantine	41100-52-1
Menadione	58-27-5
Mepenzolate	76-90-4
Mepiroxol	6968-72-5
Mepivacaine	1722-62-9

Meptazinol	59263-76-2
Mequinol	150-76-5
Mercaptopurine	50-44-2
Meropenem	96036-03-2
Mesalamine	89-57-6
Mesna	19767-45-4
Mesoridazine	32672-69-8
Mestranol	72-33-3
Metaproterenol	5874-97-5
Metaraminol	33402-03-8
Methazolamide	554-57-4
Methazolastone	85622-93-1
Methenamine	100-97-0
Methimazole	60-56-0
Methocarbamol	532-03-6
Methoxsalen	298-81-7
Methscopolamine	155-41-9
Methyclothiazide	135-07-9
Methylprednisolone	83-43-2
Methylthiouracil	56-04-2
Meticrane	1084-65-7
Metolazone	17560-51-9
Metoprolol	392-17-7
Metronidazole	443-48-1
Mevastatin	73573-88-3
Mexiletine	31828-71-4
Mianserin	21535-47-7
Mifepristone	84371-65-3
Miglitol	72432-03-2
Milnacipran	101152-94-7
Milrinone	78415-72-2
Mirabegron	223673-61-8
Mirtazapine	85650-52-8
Mitotane	53-19-0
Mitoxantrone	70476-82-3
Moclobemide	71320-77-9
Moexipril	82586-52-5
Moguisteine	119637-67-1
Mometasone	83919-23-7
Monobenzone	103-16-2
Montelukast	151767-02-1
Moroxydine	3160-91-6
Mosapride	112885-42-4
Moxonidine	75438-57-2
Mycophenolate	128794-94-5
Mycophenolic	24280-93-1
Nabumetone	42924-53-8
Nafamostat	82956-11-4
Naftopidil	57149-07-2
Nalidixic	389-08-2
Nalmefene	58895-64-0
Naloxone	357-08-4
Naltrexone	16676-29-2
Naphazoline	550-99-2
Naproxen	26159-34-2
Naratriptan	143388-64-1
Natamycin	7681-93-8
Nateglinide	105816-04-4
Nefiracetam	77191-36-7
Nelarabine	121032-29-9
Nelfinavir	159989-65-8
Nepafenac	78281-72-8
Nevirapine	129618-40-2
Niacin	59-67-6
Nialamide	51-12-7
Nicardipine	54527-84-3
Nicorandil	65141-46-0
Nicotinamide	98-92-0
Nicotine	65-31-6
Nifedipine	21829-25-4
Nifenazone	2139-47-1
Niflumic	4394-00-7
Nilotinib	641571-10-0
Nilvadipine	75530-68-6
Nimesulide	51803-78-2
Nimodipine	66085-59-4
Nisoldipine	63675-72-9
Nitazoxanide	55981-09-4
Nitrendipine	39562-70-4
Nitrofurazone	59-87-0
Nizatidine	76963-41-2
Noradrenaline	108341-18-0
norethindrone	68-22-4
Noscapine	912-60-7
Nystatin	1400-61-9
Olanzapine	132539-06-1
Olmesartan	144689-63-4
Olopatadine	140462-76-6
olsalazine	6054-98-4
Omeprazole	73590-58-6
Ondansetron	99614-01-4
Orlistat	96829-58-2
Ornidazole	16773-42-5
Orphenadrine	4682-36-4
OSI-420	183320-51-6
Ospemifene	128607-22-7
Ouabain	630-60-4
Oxaliplatin	61825-94-3
Oxaprozin	21256-18-8
Oxcarbazepine	28721-07-5
Oxeladin	52432-72-1
Oxfendazole	53716-50-0
Oxybuprocaine	5987-82-6
Oxybutynin	5633-20-5
Oxybutynin	1508-65-2
Oxymetazoline	1491-59-4
Oxytetracycline	79-57-2
Ozagrel	82571-53-7
Ozagrel	78712-43-3
Paclitaxel	33069-62-4
Paeoniflorin	23180-57-6
Pancuronium	15500-66-0
Paroxetine	78246-49-8
Pasiniazid	2066-89-9
Pazopanib	444731-52-6
Pazopanib	635702-64-6
PCI-32765	936563-96-1
Penciclovir	39809-25-1
Pentamidine	140-64-7
Pentoxifylline	1677687
Pergolide	66104-23-2
Phenacetin	62-44-2
Phenazopyridine	136-40-3
Phenformin	834-28-6

Phenindione	83-12-5
Pheniramine	132-20-7
Phenothrin	26002-80-2
Phenoxybenzamine	63-92-3
Phentolamine	65-28-1
Phenylbutazone	50-33-9
Phenylephrine	61-76-7
Phenytoin	57-41-0
Phenytoin	630-93-3
Phthalylsulfacetamide	131-69-1
Pidotimod	121808-62-6
Pilocarpine	54-71-7
Pimecrolimus	137071-32-0
Pimobendan	74150-27-9
Pimozide	2062-78-4
Pioglitazone	111025-46-8
Pioglitazone	112529-15-4
Piromidic	19562-30-2
Piroxicam	36322-90-4
Pitavastatin	147526-32-7
Pizotifen	15574-96
PMSF	329-98-6
Pomalidomide	19171-19-8
Ponatinib	943319-70-8
Posaconazole	171228-49-2
Potassium	7681-11-0
Pralatrexate	146464-95-1
Pramipexole	104632-26-0
Pramipexole	191217-81-9
Pramiracetam	68497-62-1
Pramoxine	637-58-1
Pranlukast	103177-37-3
Pranoprofen	52549-17-4
Prasugrel	150322-43-3
Pravastatin	81131-70-6
Praziquantel	55268-74-1
Prednisolone	50-24-8
Prednisolone	52-21-1
Prednisone	53-03-2
Pregnenolone	145-13-1
Pridinol	6856-31-1
Prilocaine	721-50-6
Primaquine	63-45-6
Primidone	125-33-7
Proadifen	62-68-0
Probenecid	57-66-9
Probucol	23288-49-5
Procaine	51-05-8
Prochlorperazine	84-02-6
Procodazole	23249-97-0
Procyclidine	1508-76-5
Progesterone	57-83-0
Propafenone	34183-22-7
Proparacaine	499-67-2
Propranolol	318-98-9
Propylthiouracil	51-52-5
Protionamide	14222-60-7
Pyrazinamide	98-96-4
Pyridostigmine	101-26-8
Pyridoxine	58-56-0
Pyrilamine	59-33-6
Pyrimethamine	58-14-0
Quetiapine	111974-72-2
Quinapril	82586-55-8
Quinine	6119-47-7
Racecadotril	81110-73-8
Ractopamine	90274-24-1
Raltegravir	518048-05-0
Ramelteon	196597-26-9
Ramipril	87333-19-5
Ranitidine	66357-59-3
Ranolazine	95635-55-5
Ranolazine	95635-56-6
Rapamycin	53123-88-9
Rasagiline	161735-79-1
Rebamipide	90098-04-7
Reboxetine	98769-84-7
Regorafenib	755037-03-7
Repaglinide	135062-02-1
Reserpine	50-55-5
Resveratrol	501-36-0
Ribavirin	36791-04-5
Riluzole	1744-22-5
Rimantadine	13392-28-4
Rimonabant	168273-06-1
Risperidone	106266-06-2
Ritodrine	23239-51-2
Ritonavir	155213-67-5
Rivaroxaban	366789-02-8
Rivastigmine	129101-54-8
Rizatriptan	145202-66-0
Rocuronium	119302-91-9
Rofecoxib	162011-90-7
Roflumilast	162401-32-3
Rolipram	61413-54-5
Ronidazole	7681-76-7
Ropinirole	91374-20-8
Ropivacaine	98717-15-8
Rosiglitazone	122320-73-4
Rosiglitazone	302543-62-0
Rosiglitazone	155141-29-0
Rosuvastatin	147098-20-2
Rotigotine	99755-59-6
Roxatidine	93793-83-0
Roxithromycin	80214-83-1
Rufinamide	106308-44-5
Ruxolitinib	941678-49-5
Rolipram	85416-73-5
Salicylanilide	87-17-2
Sasapyrine	552-94-3
Saxagliptin	361442-04-8
Scopine	498-45-3
Scopolamine	114-49-8
Secnidazole	3366-95-8
Serotonin	153-98-0
Sertaconazole	99592-39-9
Sertraline	79559-97-0
Sildenafil	171599-83-0
Silodosin	160970-54-7
Simvastatin	79902-63-9
Sodium	94-16-6
Sodium	134-03-2
Sodium	7632-00-0
Sodium	14402-89-2

Picosulfate	10040-45-6
Sodium	54-21-7
Solifenacin	242478-38-2
Sorbitol	50-70-4
Sotalol	959-24-0
Spectinomycin	21736-83-4
Spiramycin	8025-81-8
Spironolactone	52-01-7
Stavudine	3056-17-5
Streptozotocin	18883-66-4
Sucralose	56038-13-2
Sulbactam	68373-14-8
Sulbactam	69388-84-7
sulfacetamide	127-56-0
Sulfadiazine	68-35-9
Sulfaguanidine	57-67-0
Sulfamerazine	127-79-7
Sulfameter	651-06-9
Sulfamethazine	57-68-1
Sulfamethizole	144-82-1
Sulfanilamide	63-74-1
Sulfasalazine	599-79-1
Sulindac	38194-50-2
Sumatriptan	103628-48-4
Sunitinib	341031-54-7
Suplatast	94055-76-2
Suprofen	40828-46-4
Tacrine	1684-40-8
Tadalafil	171596-29-5
TAME	901-47-3
Tazarotene	118292-40-3
Telaprevir	402957-28-2
Telbivudine	3424-98-4
Telmisartan	144701-48-4
Temocapril	110221-44-8
Temsirolimus	162635-04-3
Tenofovir	147127-20-6
Tenofovir	202138-50-9
Tenoxicam	59804-37-4
Terazosin	70024-40-7
Terbinafine	91161-71-6
Terbinafine	78628-80-5
Teriflunomide	108605-62-5
Tetracaine	136-47-0
tetrahydrozoline	522-48-5
Thalidomide	50-35-1
Thiabendazole	148-79-8
Tianeptine	30123-17-2
Tigecycline	220620-09-7
Tilmicosin	108050-54-0
tinidazole	19387-91-8
Tiopronin	19392
Tiotropium	139404-48-1
Tioxolone	4991-65-5
Tiratricol	51-24-1
Tizanidine	64461-82-1
Tofacitinib	540737-29-9
Tolbutamide	64-77-7
Tolfenamic	13710-19-5
Tolmetin	64490-92-2
Tolnaftate	2398-96-1
Tolperisone	3644-61-9
Tolterodine	124937-52-6
toltrazuril	69004-03-1
Tolvaptan	150683-30-0
Topiramate	97240-79-4
Topotecan	119413-54-6
Tranilast	53902-12-8
Tretinoin	302-79-4
Triamcinolone	124-94-7
Triamcinolone	76-25-5
triamterene	396-01-0
Trichlormethiazide	133-67-5
Triclabendazole	68786-66-3
Trifluoperazine	440-17-5
Triflupromazine	1098-60-8
Triflusal	322-79-2
Trilostane	13647-35-3
Trimebutine	39133-31-8
Trimipramine	521-78-8
Tripelennamine	154-69-8
Trometamol	77-86-1
Tropicamide	1508-75-4
Tropisetron	105826-92-4
Trospium	10405-02-4
Troxipide	30751-05-4
Tylosin	74610-55-2
Ulipristal	159811-51-5
Uracil	66-22-8
Urapidil	64887-14-5
Uridine	58-96-8
Ursodiol	128-13-2
Valaciclovir	124832-27-5
valganciclovir	175865-59-5
Valproic	1069-66-5
Valsartan	137862-53-4
Vandetanib	443913-73-3
Vardenafil	330808-88-3
Vecuronium	50700-72-6
Vemurafenib	918504-65-1
Venlafaxine	99300-78-4
Verteporfin	129497-78-5
Vidarabine	5536-17-4
Vildagliptin	274901-16-5
Vinblastine	143-67-9
Vincristine	2068-78-2
Vinorelbine	125317-39-7
Vismodegib	879085-55-9
Vitamin	68-19-9
Vitamin	50-81-7
Vitamin	50-14-6
Vitamin	67-97-0
Voglibose	83480-29-9
Voriconazole	137234-62-9
Vorinostat	149647-78-9
XL-184	849217-68-1
Xylazine	23076-35-9
Xylometazoline	1218-35-5
Xylose	25990-60-7
Zalcitabine	7481-89-2
Zaltoprofen	74711-43-6
Zanamivir	139110-80-8
Zidovudine	30516-87-1
Zileuton	111406-87-2

Ziprasidone	122883-93-6
Zolmitriptan	139264-17-8
Zonisamide	68291-97-4
Zoxazolamine	61-80-3
